# Cognition, Aryl Hydrocarbon Receptor Repressor Methylation, and Abstinence Duration-Associated Multimodal Brain Networks in Smoking and Long-Term Smoking Cessation

**DOI:** 10.3389/fnins.2022.923065

**Published:** 2022-07-27

**Authors:** Shile Qi, Zening Fu, Lei Wu, Vince D. Calhoun, Daoqiang Zhang, Stacey B. Daughters, Ping-Ching Hsu, Rongtao Jiang, Victor M. Vergara, Jing Sui, Merideth A. Addicott

**Affiliations:** ^1^College of Computer Science and Technology, Nanjing University of Aeronautics and Astronautics, Nanjing, China; ^2^Tri-Institutional Center for Translational Research in Neuroimaging and Data Science (TReNDS), Georgia State University, Georgia Institute of Technology, Emory University, Atlanta, GA, United States; ^3^Department of Psychology and Neuroscience, University of North Carolina at Chapel Hill, Chapel Hill, NC, United States; ^4^Department of Environmental and Occupational Health, University of Arkansas for Medical Sciences, Little Rock, AR, United States; ^5^Department of Radiology and Biomedical Imaging, Yale University, New Haven, CT, United States; ^6^State Key Laboratory of Cognitive Neuroscience and Learning, Beijing Normal University, Beijing, China; ^7^Department of Physiology and Pharmacology, Wake Forest University School of Medicine, Winston-Salem, NC, United States

**Keywords:** smokers, ex-smokers, tobacco, neuroimaging, cessation, cognition, methylation, multimodal fusion

## Abstract

Cigarette smoking and smoking cessation are associated with changes in cognition and DNA methylation; however, the neurobiological correlates of these effects have not been fully elucidated, especially in long-term cessation. Cognitive performance, percent methylation of the aryl hydrocarbon receptor repressor (*AHRR*) gene, and abstinence duration were used as references to supervise a multimodal fusion analysis of functional, structural, and diffusion magnetic resonance imaging (MRI) data, in order to identify associated brain networks in smokers and ex-smokers. Correlations among these networks and with smoking-related measures were performed. Cognition-, methylation-, and abstinence duration-associated networks discriminated between smokers and ex-smokers and correlated with differences in fractional amplitude of low frequency fluctuations (fALFF) values, gray matter volume (GMV), and fractional anisotropy (FA) values. Long-term smoking cessation was associated with more accurate cognitive performance, as well as lower fALFF and more GMV in the hippocampus complex. The methylation- and abstinence duration-associated networks positively correlated with smoking-related measures of abstinence duration and percent methylation, respectively, suggesting they are complementary measures. This analysis revealed structural and functional co-alterations linked to smoking abstinence and cognitive performance in brain regions including the insula, frontal gyri, and lingual gyri. Furthermore, *AHRR* methylation, a promising epigenetic biomarker of smoking recency, may provide an important complement to self-reported abstinence duration.

## Introduction

Cigarette smoking continues to be a leading cause of preventable disease and death in the United States and around the world (DHHS, 2014; Reitsma et al., 2017). Smoking-attributed diseases include heart attacks, stroke, chronic obstructive pulmonary disease, diabetes, and cancer (Rostron et al., 2014). Also, smoking is associated with shorter life expectancy (Renteria et al., 2016). In addition to its primary addictive component, nicotine, cigarette smoke is composed of thousands of chemicals (Rodgman and Perfetti, 2009) that produce toxicological effects throughout the body (Talhout et al., 2011). While most individuals who smoke cigarettes in the U.S. report a desire to quit, only about 7% of quit attempts each year are successful (Babb et al., 2017), which illustrates the difficulty of quitting and remaining abstinent. However, as of 2015, 59% of U.S. adults who had ever smoked had quit (Babb et al., 2017). Smoking cessation is associated with improved pulmonary function, as well as reduced risk of stroke, heart disease, and cancer (Fagerström, 2002). Most studies compare smokers to non-smokers, however, cigarette smoke contains numerous toxins that have a wide range of effects throughout the body. Therefore, smokers and non-smokers not only differ on tobacco use disorder status, but also on the extent of exposure to tobacco-containing toxins, and perhaps also differ in pre-existing susceptibility to developing tobacco use disorder. Thus, ex-smokers may be a preferable comparator group than non-smokers. In addition, investigating the relationship between ex-smokers’ abstinence duration in relation to brain structure and function may provide insights into the beneficial effects of quitting smoking. Here, we investigated the effects of smoking on brain structure and function of current smokers compared to long-term ex-smokers.

The chemicals in cigarette smoke have two types of effects on the brain: effects specifically related to tobacco addiction and effects more generally related to long-term toxin exposure. Nicotine exerts its reinforcing effects by binding to nicotinic acetylcholine receptors (nAChR) in the mesolimbic dopamine system (Balfour et al., 2000). Chronic tobacco use results in an upregulation of nAChR and a downregulation of dopamine receptors (Storage and Brody, 2012). Changes in these and other receptor systems, which lead to changes in brain structure and function, are thought to be responsible for the classic symptoms of tobacco addiction, such as craving, tolerance, dependence, and withdrawal (Markou, 2008). Indeed, self-reported nicotine dependence severity has been associated with decreased gray matter volume (GMV) in the insula (Wang et al., 2019). Nicotine and other components of cigarette smoke also have effects on cognitive performance. Acutely, nicotine is a mild psychostimulant that can improve reaction time and accuracy (although this effect in chronic tobacco users may be due to the reversal of withdrawal symptoms) (Heishman et al., 2010; Mcclernon et al., 2015). However, cigarette smoke consists of numerous compounds associated with brain toxicity that can result in inflammation, atherosclerosis, white matter hyperintensities, and brain atrophy, all of which can negatively impact cognitive function over time (Swan and Lessov-Schlaggar, 2007). Chronic cigarette smokers typically perform worse on measures of attentional control, inhibition, memory, and information processing speed compared to age-, sex-, and education level-matched non-smokers (Hill et al., 2003; Chamberlain et al., 2012; Almeida et al., 2020). Evidence suggests that nicotinic receptor systems return to non-smoker levels following smoking cessation (Storage and Brody, 2012) and ex-smokers demonstrate better cognitive performance compared to smokers (Ernst et al., 2001; Kalmijn et al., 2002). While smoking is well known to negatively affect the brain and cognition, the functional and structural brain correlates of cognitive performance differences among smokers and ex-smokers is largely unknown.

Recently, DNA methylation profiles have emerged as a promising new epigenetic biomarker of cigarette exposure. DNA methylation is a common epigenetic signaling tool that cells use to regulate gene activation and expression. Cigarette smoking is associated with extensive genome-wide differences in DNA methylation, in particular, smoking is associated with hypomethylation of the cg05575921 loci in the aryl hydrocarbon receptor repressor (*AHRR*) gene (Philibert et al., 2013; Zeilinger et al., 2013; Gao et al., 2015; Ambatipudi et al., 2016). The *AHRR* gene regulates the aryl hydrocarbon receptor (AHR), which is the induction point for the xenobiotic pathway responsible for the degradation of environmental toxins commonly found in cigarettes. The hypomethylation of the *AHRR* gene shown in smokers likely represents increased AHR activation of this pathway from smoking exposure (summarized in Philibert et al., 2013) and smoking cessation is associated with more *AHRR* methylation (Zeilinger et al., 2013; Philibert et al., 2016). Indeed, *AHRR* methylation has been proposed as a biomarker of smoking and smoking cessation (Philibert et al., 2013, 2016). DNA methylation profiles derived from blood and saliva/buccal cells have been associated with changes in brain structure and function (Wheater et al., 2020). To our knowledge, there has only been a single published study linking tobacco smoking-related differences in methylation with brain structure and other smoking-related health outcomes (Corley et al., 2019). Higher values of smoking-related methylation scores were associated with lower cognitive function and thinner cortical gray matter across a network of brain regions (Corley et al., 2019). Corley et al. (2019) indicated that methylation patterns accounted for more variance in smoking-related morbidities than phenotypic self-reports (e.g., smoking status and pack years). Therefore, methylation biomarkers may provide an important complement to self-reported smoking history because such biomarkers are not affected by recall bias and may be more sensitive measures of smoking-related disease risk (Corley et al., 2019). For this reason, we investigated both abstinence duration and *AHRR* methylation, hypothesizing that *AHRR* methylation would be more informative of differences in brain structure/function. However, it is unknown whether *AHRR* methylation-associated differences in brain structure/function among smokers and ex-smokers are unique, or whether they overlap with differences associated with smoking abstinence duration.

The present study uses a supervised multimodal fusion analysis to investigate structural and functional brain networks that can discriminate between current and ex-smokers and are associated with cognitive performance, DNA methylation, and abstinence duration. This data-driven approach combined three complementary imaging modalities: gray matter volume (GMV) from structural magnetic resonance imaging (sMRI), fractional anisotropy (FA) values from diffusion MRI (dMRI), and fractional amplitude of low frequency fluctuation (fALFF) from resting-state functional MRI (rs-fMRI). Smoking is associated with reduced GMV and FA values, which is indicative of the negative effects of smoking on the brain (Gray et al., 2020). fALFF is a physiological variable derived from the blood oxygen level dependent (BOLD) signal and has been shown to correspond with the metabolic rate of glucose and oxygen in different brain regions (Deng et al., 2022). Smoking has been associated with higher fALFF values in reward-related brain regions (Weng et al., 2021). Altogether, GMV, FA, and fALFF can provide insights into how smoking affects brain structure and metabolism. While most studies investigate a single modality, or analyze multiple modalities independently from one another, the benefit of the supervised fusion is that all the variables are estimated jointly. This ensures that all the regions in an identified network covary together and also correlate with the targeted clinical variables, thus improving interpretability (Qi et al., 2018a). Furthermore, fusion analyses can identify relationships among large, inter-related datasets (e.g., brain structure and function data) while adjusting for multiple comparisons, in order to reveal the most relevant variables (Calhoun and Sui, 2016). In this sample of smokers and long-term ex-smokers, we focused on the following aims: (1) to determine the structural and functional brain patterns associated with cognitive performance; (2) to determine the brain patterns associated with an *AHRR* methylation biomarker of smoking exposure; (3) to determine the brain patterns associated with the duration of the most recent quit attempt; and (4) to evaluate the relationships between cognitive performance, *AHRR* methylation, abstinence duration, and other smoking-related measures. These results could help inform how these separate, but related, variables are affected by smoking and long-term smoking cessation.

## Materials and Methods

### Participants

Current and former tobacco users were recruited from the Little Rock, AR community, including *N* = 29 current smokers, *N* = 30 ex-smokers (abstinent from all tobacco/nicotine > 2 years). Participants were aged 25–55 years; the lower age limit minimized potential age differences between groups (considering the ≥2 year abstinence criteria for ex-smokers), and the upper age limit reduced the likelihood of age-related changes in cognition. Smokers reported smoking ≥ 7 cigarettes/day for ≥2 years and had an expired breath carbon monoxide (CO) concentration of ≥5 ppm (Vitalograph Inc., Lenexa, KS, United States). Smokers were excluded if they reported daily use of other tobacco products (e.g., little cigars or electronic cigarettes). Ex-smokers reported smoking ≥ 7 cigarettes/day for ≥2 years, but reported no use of tobacco or nicotine ≥ 2 years, and had breath CO ≤ 5 ppm. The ex-smoker abstinence duration criterion minimized the likelihood of future relapse (Wetter et al., 2004; Hawkins et al., 2010). Participants were excluded if they met any of the following criteria: (1) reported serious health problems, (2) had a history of head trauma or neurological disorders, (3) currently met criteria for an Axis I psychiatric disorder (based on a MINI Neuropsychiatric Interview) (Sheehan, 2016), (4) reported heavy drug use or problems with drugs or alcohol in the past 6 months (other than tobacco), (5) had a positive urine test for drugs (i.e., amphetamine, cocaine, methamphetamine, opioids, benzodiazepines, and barbiturates) or breath test for alcohol, (6) reported using cannabis more than 4 days a week or more than 2 g per week, (7) were pregnant, (8) were using psychoactive medications other than first-line medications for depression (e.g., sertraline), (9) had less than a 9th grade education, (10) weighed more than 350 pounds (due to the weight limit of the MRI scanner), (11) or could not achieve 70% accuracy in the Paced Auditory Serial Addition Test (PASAT) easy practice task during eligibility screening. Participants were allowed to play the task up to three times to meet criterion (details can be found in Supplementary Material). Participants provided written informed consent and this study was approved by the University of Arkansas for Medical Sciences Institutional Review Board and conducted in accordance with the Declaration of Helsinki and relevant institutional guidelines and policies.

### Methods

During eligibility screening, participants were administered a tobacco use history structured interview that assessed the current and lifetime use of cigarettes and other tobacco products, including the number and duration of cessation attempts, DSM-5 tobacco use disorder (TUD) severity scores (APA, 2013), and the Fagerstrom Test for Nicotine Dependence (FTND) (Heatherton et al., 1991). Cigarettes per day, TUD, and FTND scores for ex-smokers were based on their past smoking behavior. If eligible, participants were scheduled for a 2-h study session including a 1-h MRI scan and a 1-h behavioral testing session. Smokers were instructed to smoke immediately prior to the study visit and the time of their last cigarette was recorded. During the MRI scan, the order of scans was sMRI, rs-fMRI, dMRI, and PASAT fMRI (details on imaging parameters and preprocessing steps can be found in Supplementary Material). The PASAT is a mental arithmetic task that requires participants to mentally sum numbers sequentially as they appear onscreen and select the correct sum from an array of options before the next number appears. Following the MRI scan, participants submitted a saliva sample for DNA testing. Participants were allowed to opt-out of submitting a saliva sample and remain in the study. Participants were compensated up to $178 for completion of the study.

Saliva samples were tested for DNA methylation using the illumina Infinium Methylation EPIC 850K BeadChip Kit, which interrogates over 850,000 methylation loci quantitatively across the genome at single-nucleotide resolution. In particular, the methylation loci cg05575921 for the aryl hydrocarbon receptor repressor (*AHRR*) gene, which plays an important role in inflammation, cell differentiation, and cell cycle control, has previously been associated with smoking status and smoking intensity (Zeilinger et al., 2013; Ambatipudi et al., 2016; Philibert et al., 2020). Percent methylation of the cg05575921 loci is used here as a biological marker of recency and heaviness of cigarette smoking, and was significantly higher among ex-smokers compared with smokers (FDR *p* = 2.9e-08). Based on the Receiver Operating Characteristic (ROC) analysis, cg05575921 also presented with the strongest predictive accuracy (AUC = 0.97) for the classification of smokers versus ex-smokers (complete details regarding the PASAT and saliva DNA methylation analysis can be found in the Supplementary Material).

Table 1 shows the demographic characteristics and tobacco use histories for smokers and ex-smokers. Groups were similar in age, sex distribution, ethnicity, and years of education (*p*’s > 0.05). However, the smoker group had more African American participants (*p* = 0.006) (the primary analyses remained significant after correcting for race, see Supplementary Material). Smokers’ current FTND and DSM-5 TUD scores were similar to ex-smokers’ past scores (*p*’s > 0.05). Ex-smokers smoked more cigarettes per day (*p* = 0.022), although the number of pack years was similar between groups (*p* = 0.10). As expected, ex-smokers had lower breath CO concentration (*p* < 0.001) and the duration of their most recent quit attempt was longer than smokers’ (*p* < 0.001). Ex-smokers quit smoking an average of 8.9 years ago (range 2–21 years).

**TABLE 1:** Demographic characteristics and tobacco use histories of smokers and ex-smokers (M ± SD).

	Smokers	Ex-smokers	Group difference
Sample size	*N* = 29	*N* = 30	
Age	38.8±9.9	40.5±7.6	*p* = 0.46
Gender (M/F)	17/12	11/19	*p* = 0.09
Race (White/African/American/Asian/other)	19/8/1/1	30/0/0/0	χ^2^(3) = 12.5, *p* = 0.006
Ethnicity (non-hispanic/hispanic)	29/0	30/0	N.S.
Years of education	14.0±3.0	14.7±2.4	*p* = 0.36
FTND	4.8±2.1	5.1±2.1 (past)	*p* = 0.63
DSM-5 TUD	6.0±2.4	6.7±2.2 (past)	*p* = 0.23
Cigarettes per day	17.6±7.0	22.1±7.8 (past)	T(57) = 2.4, *p* = 0.022
Pack years	22.9±22.2	15.4±9.0	*p* = 0.10
Duration of most recent quit attempt (days)	98.5±342.2	3,262.3±2,143.1	T(57) = 7.9, *p* < 0.001
Breath CO (ppm)	26.1±14.9	2.3±1.3	T(57) = 8.7, *p* < 0.001
PASAT accuracy (% correct)	73.2±11.0	81.6±9.1	T(57) = 3.2, *p* = 0.002
Mean FD (head motion)	0.25±0.11	0.25±0.12	*p* = 0.81
Sample size (saliva samples)	*N* = 21	*N* = 26	
cg05575921 loci (% methylation)	0.50±0.09	0.73±0.06	T(45) = 10.1, *p* < 0.001

*FTND, Fagerstrom Test of nicotine dependence; DSM-5 TUD, DSM-5 tobacco use disorder; PASAT, Paced Auditory Serial Addition Test; Mean FD, mean frame-wise displacements.*

Paced Auditory Serial Addition Test accuracy (% correct) was lower among smokers compared to ex-smokers (*p* = 0.002). Twenty-one smokers and 26 ex-smokers submitted saliva samples. The *AHRR* cg05575921 loci was less methylated among smokers compared to ex-smokers (*p* < 0.001; Table 1). Across all participants, there was a positive correlation between PASAT accuracy and cg05575921 percent methylation (*r* = 0.31, *p* = 0.033), and there was a positive correlation between cg05575921 percent methylation and abstinence duration (*r* = 0.65, *p* < 0.001). rs-fMRI, sMRI, and dMRI data were collected (details can be found in Supplementary Material) and fALFF from fMRI, GMV from sMRI and FA from dMRI were used as fusion analysis input. Head motion during scanning (measured as mean frame-wise displacements) was similar between groups (Table 1).

### Analysis Pipeline

**FIGURE 1:**
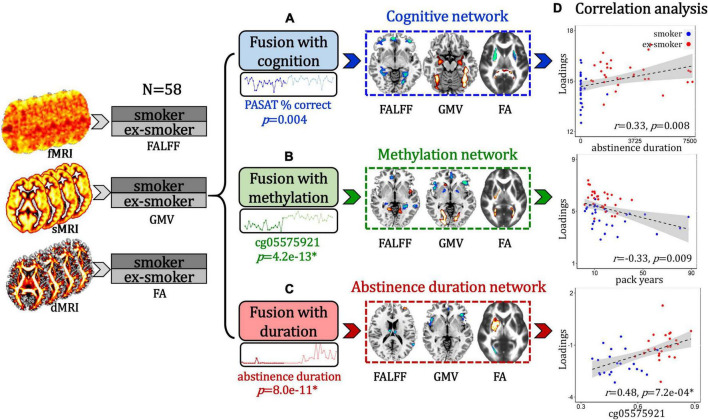
Flow diagram of the study design. **(A)** Paced Auditory Serial Addition Test (PASAT)-guided fusion was performed on fALFF + GMV + FA to identify a smoker and ex-smoker group-discriminating multimodal brain network that associated with cognition. **(B)** cg05575921 loci methylation-guided fusion was used to identify a smoker and ex-smoker group-discriminating multimodal brain network that associated with DNA methylation. **(C)** Duration of the last quit attempt-guided fusion was used to identify an abstinence duration-associated multimodal brain network that discriminated between smokers and ex-smokers. **(D)** Correlation analyses were performed between these identified multimodal networks and smoking-related measures.

**FIGURE 2:**
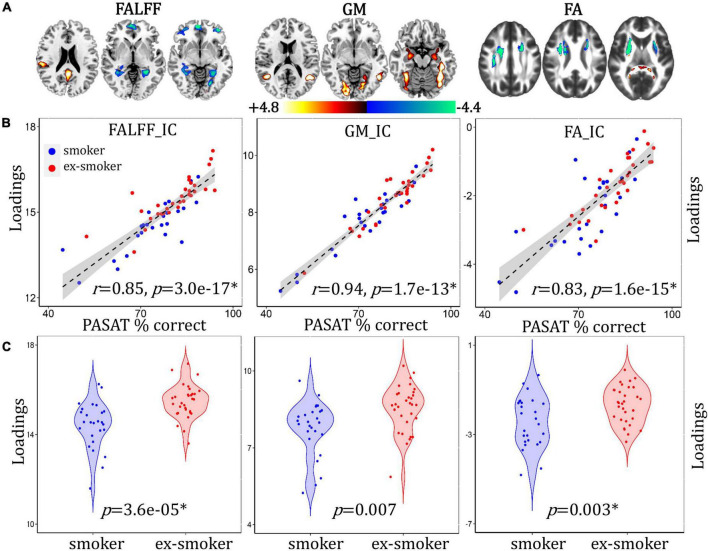
Cognition-associated fALFF + GMV + FA multimodal joint components. **(A)** The spatial brain independent component (IC) networks visualized at |Z| > 2.5. Regions where smokers > ex-smokers indicated by blue color, regions where ex-smokers > smokers indicated by red color. **(B)** Correlations between components’ loadings and PASAT% correct. **(C)** Group difference of the components among smokers and ex-smokers.

**FIGURE 3:**
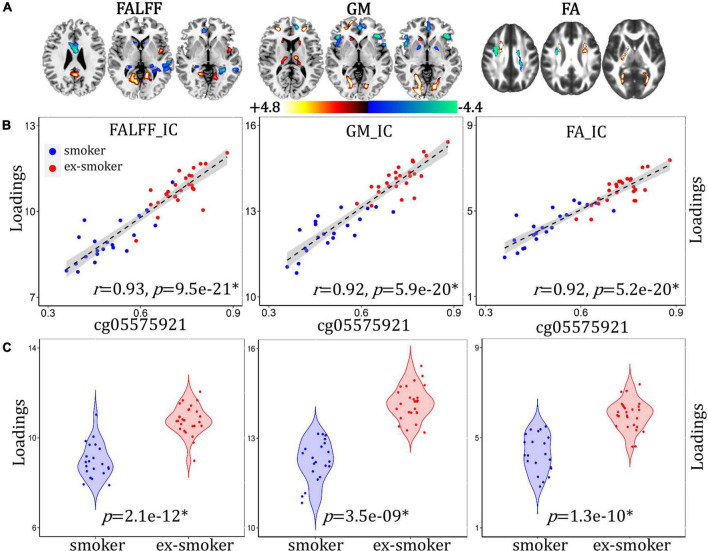
Methylation-associated fALFF + GMV + FA multimodal joint components. **(A)** The spatial brain independent component (IC) networks visualized at |Z| > 2.5. Regions where smokers > ex-smokers indicated by blue color, regions where ex-smokers > smokers indicated by red color. **(B)** Correlations between components’ loadings and cg05575921 loci methylation. **(C)** Group difference of the components among smokers and ex-smokers.

**FIGURE 4:**
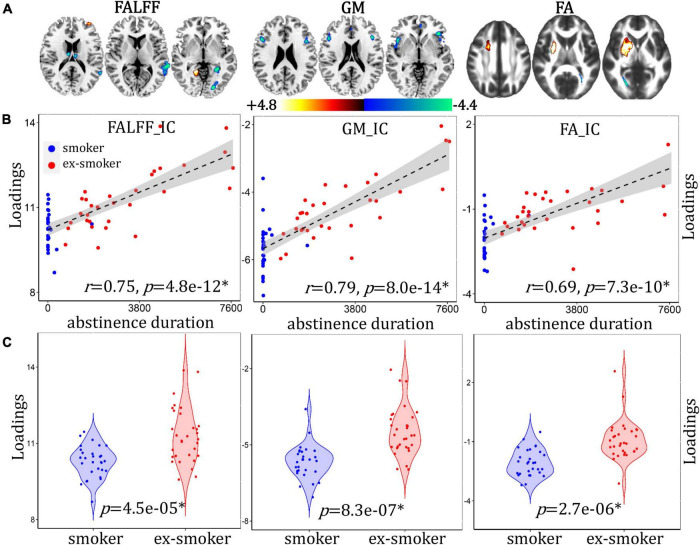
Abstinence duration-associated fALFF + GMV + FA multimodal joint components. **(A)** The spatial brain independent component (IC) networks visualized at |Z| > 2.5. Regions where smokers > ex-smokers indicated by blue color, regions where ex-smokers > smokers indicated by red color. **(B)** Correlations between components’ loadings and abstinence duration. **(C)** Group difference of the components among smokers and ex-smokers.

To accomplish the goals stated in the introduction, we performed the following analyses: (1) PASAT-guided fusion was used to identify a cognition-associated multimodal brain network (Figure 1A); (2) cg05575921 loci methylation-guided fusion was used to identify a DNA methylation-associated multimodal brain network (Figure 1B); (3) Duration of the last quit attempt-guided fusion was used to identify an abstinence duration-associated multimodal brain network (Figure 1C); (4) Correlation analyses were performed between these identified multimodal networks and smoking-related measures (Figure 1D).

Specifically, subject-wise PASAT performance/cg05575921 loci methylation/abstinence duration values were used as a reference to jointly decompose the preprocessed fALFF (*X*_1_), GMV (*X*_2_), and FA (*X*_3_) by “MCCAR + jICA” (Qi et al., 2018a,b, 2021) (multi-site canonical correlation analysis with reference + joint independent component analysis) to investigate cognition/methylation/abstinence duration-associated multimodal brain networks that differed between smokers and ex-smokers. This data-driven method decomposes features of each modality into spatial maps and their corresponding canonical variants, then seeks to identify co-varying brain patterns among the three modalities by maximizing the inter-modality correlations between canonical variants. For each modality matrix *X*_i_, fALFF/GMV/FA features were stacked as in Eq. (1).


(1) $$X_{\text{i}}=\left\{\begin{array}{c} X_{\text{smoker}}\\ X_{\text{ex}-\text{smoker}} \end{array}\right\}$$

The correlations of imaging components with clinical measures of interest (*i.e.*, PASAT performance/cg05575921 loci methylation/abstinence duration) was maximized in the supervised fusion method, as in Eq. (2).


(2) $$\text{max}\,\sum_{\text{k},\text{j}=1}^{2}\left\{{||\text{corr}\,\left(A_{\text{k}},A_{\text{j}}\right)||}_{2}^{2}+2\,\lambda \cdot {||\text{corr}\,\left(A_{\text{k}},r\,e\,f\right)||}_{2}^{2}\right\}$$

where *A*_*k*_ is the mixing matrix for each modality, corr(*A*_k_,*A*_j_) is the column-wise correlation between *A*_k_ and *A*_j_, and*corr*(*A*_k_,*ref*) is the column-wise correlation between *A*_*k*_ and the reference signal (PASAT performance/cg05575921 loci methylation/abstinence duration). This supervised fusion method can extract a joint multimodal component(s) that correlate with PASAT performance/cg05575921 loci methylation/abstinence duration.

Correlation analyses were performed to evaluate the relationship between cognition/methylation/duration-associated multimodal networks and smoking-related measures. To make comparable comparisons among cognition, methylation, and abstinence duration-associated multimodal brain networks, the same independent component number (*M* = 30) and the same λ (λ = 1) were used in all the above reference-guided fusion analyses.

## Results

### Cognition-Associated Network Differentiating Between Smoker and Ex-smoker Groups

A cognition-associated multimodal component was identified that correlated with PASAT% correct (Figure 2B, for fALFF, *r* = 0.85, *p* = 3.0e-17*; for GMV, *r* = 0.94, *p* = 1.7e-13*; for FA, *r* = 0.83, *p* = 1.6e-15*) and discriminated between smoker and ex-smoker groups (Figure 2C, for fALFF, *p* = 3.6e-05*; for GM, *p* = 0.007; for FA, *p* = 0.003*). The brain areas (Figure 2A) in the cognition-associated network are summarized in Supplementary Table 1. In general, the spatial brain network maps and the loadings are orthogonal to one another. Higher group loadings associated with negative values on the brain maps suggest that subjects with higher loadings have a subtractive effect on the neuroimaging data. The correlations remain significant after regressing out age, race, years of education and mean FD (for fALFF, *r* = 0.84, *p* = 3.8e-15*; for GM, *r* = 0.93, *p* = 6.7e-13*; for FA, *r* = 0.80, *p* = 3.7e-13*). Compared with current smokers, ex-smokers had higher fALFF values in the bilateral posterior cingulate cortex (PCC) and middle temporal gyrus (MTC), and lower fALFF values in the bilateral superior/middle frontal gyrus (S/MFG), parahippocampal gyrus (PHG), lingual gyrus (LG), and anterior cingulate cortex (ACC). Ex-smokers also had more GMV in the bilateral LG, fusiform gyrus (FG), superior/middle temporal gyrus (S/MTG), and PHG. Lastly, ex-smokers had higher FA values in the bilateral forceps major and lower FA values in the bilateral superior longitudinal fasciculus (SLF) and anterior thalamic radiation (ATR).

### Methylation-Associated Network Differentiating Between Smoker and Ex-smoker Groups

A methylation-associated multimodal component was identified that correlated with cg05575921 loci percent methylation (Figure 3B, for fALFF, *r* = 0.93, *p* = 9.5e-21*; for GM, *r* = 0.92, *p* = 5.9e-20*; for FA, *r* = 0.92, *p* = 5.2e-20*) and discriminated between smoker and ex-smoker groups (Figure 3C, for fALFF, *p* = 2.1e-12*; for GM, *p* = 3.5e-09*; for FA, *p* = 1.3e-10*). The brain areas (Figure 3A) in the methylation-associated network are summarized in Supplementary Table 2. The correlations remain significant after regressing out age, race, years of education and mean FD (for fALFF, *r* = 0.93, *p* = 9.7e-21*; for GM, *r* = 0.92, *p* = 7.1e-20*; for FA, *r* = 0.91, *p* = 1.1e-19*). Compared with current smokers, ex-smokers had higher fALFF values in the bilateral LG, PCC, FG, and insula, and lower fALFF values in the bilateral S/MTG, ACC, MFG, caudate, and PHG. Ex-smokers also had more GMV in the bilateral LG, FG, S/MFG, thalamus, and left caudate, and less GMV in the bilateral middle/inferior frontal gyrus (M/IFG), ACC, and insula. Lastly, ex-smokers had higher FA values in the middle part of the bilateral forceps major, and lower FA values in the bilateral ATR and SLF.

### Abstinence Duration-Associated Network Differentiating Between Smoker and Ex-smoker Groups

An abstinence duration-associated multimodal component was identified that correlated with duration of the last quit attempt (Figure 4B, for fALFF, *r* = 0.75, *p* = 4.8e-12*; for GMV, *r* = 0.79, *p* = 8.0e-14*; for FA, *r* = 0.69, *p* = 7.3e-10*) and discriminated between smoker and ex-smoker groups (Figure 4C, for fALFF *p* = 5.1e-05*; for GM, *p* = 8.3e-07*; for FA, *p* = 2.7e-06*). The brain areas (Figure 4A) in the abstinence duration-associated network are summarized in Supplementary Table 3. The correlations remain significant after regressing out age, race, years of education and mean FD (for fALFF, *r* = 0.74, *p* = 4.1e-12*; for GM, *r* = 0.75, *p* = 7.0e-14*; for FA, *r* = 0.64, *p* = 4.5e-10*). Compared with current smokers, ex-smokers had higher fALFF values in the bilateral LG and MFG, and lower fALFF values in the bilateral S/MTG, LG, PHG, and thalamus. Ex-smokers also had less GMV in the bilateral inferior/MFG and insula. Lastly, ex-smokers had higher FA values in the bilateral ATR and SLG, and lower FA values in the posterior part of the bilateral forceps major.

### Relationship With Smoking-Related Measures

**FIGURE 5:**
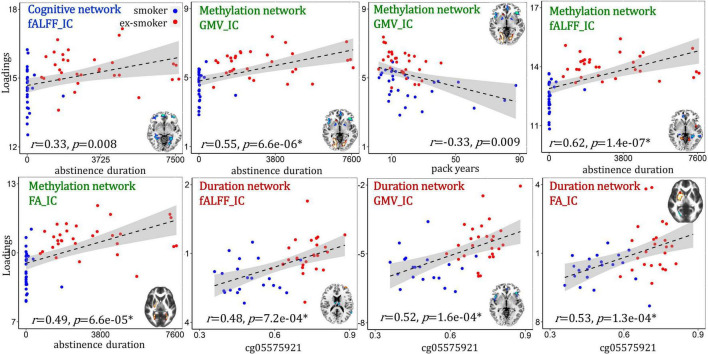
Correlations between the identified multimodal brain networks and smoking-related measures.

We tested the correlations between the identified cognition/methylation/abstinence duration-associated networks with smoking-related measures (i.e., cg05575921 percent methylation, duration of most recent quit attempt and pack years, Figure 5). A conservative significance threshold for multiple comparisons was applied (B-Y method FDR correction, alpha for 9 hypothesis tests = 0.018) (Narum, 2006). For the cognition-associated network, fALFF_IC (IC: independent component) positively correlated with the duration of the most recent quit attempt (*r* = 0.33, *p* = 0.008), *i.e.*, higher IC loadings associated with longer abstinence durations. For the methylation-associated network, fALFF_IC positively correlated with abstinence duration (*r* = 0.62, *p* = 1.4e-07*); GMV_IC positively correlated with abstinence duration (*r* = 0.55, *p* = 6.6e-06*) and negatively correlated with pack years (*r* = –0.33, *p* = 0.009); and FA_IC positively correlated with abstinence duration (*r* = 0.49, *p* = 6.6e-05*). For the abstinence duration-associated network, fALFF_IC, GMV_IC and FA_ICA were all positively correlated with the percent methylation of the cg05575921 loci (*r* = 0.48, *p* = 7.2e-04*; *r* = 0.52, *p* = 1.6e-04*; *r* = 0.53, *p* = 1.3e-04*, respectively).

## Discussion

This study investigated differences between current smokers’ and ex-smokers’ brain structure and function using supervised multimodal fusion analysis. This fusion approach can reveal group differences that covary across MRI modalities. We identified brain networks related to smoker/ex-smoker differences in cognition, *AHRR* methylation, and abstinence duration. This is among the first neuroimaging study to compare smokers to long-term ex-smokers. Unlike non-smokers, ex-smokers have been exposed to the same toxins and have the same susceptibility to tobacco use disorder as smokers. Indeed, our sample of smokers and ex-smokers had a similar number of pack years, making the ex-smokers a good comparator group in terms of lifetime smoke exposure. The results indicated there are differences in brain structure/function uniquely associated with better cognitive accuracy, *AHRR* methylation, and abstinence duration among individuals who successfully quit smoking. In particular, long-term smoking cessation was associated with more accurate cognitive performance, as well as lower fALFF and more GMV in the hippocampus complex. Furthermore, the methylation- and abstinence duration-associated networks overlapped and positively correlated with abstinence duration and percent methylation, respectively, suggesting that *AHRR* methylation complements self-reported abstinence duration.

Supervised multimodal fusion analyses have been previously applied to identify differences between individuals with (*n* = 35) and without (*n* = 37) cocaine use disorder (CUD) using a measure of delay discounting as the *a priori* reference (Meade et al., 2021). Components were negatively correlated with delay discounting and individuals with CUD had lower component loadings for all three MRI modalities (GM volume, FA values, and regional homogeneity of resting-state fMRI). These components related to structural and functional co-alterations in regions involved in reward salience, executive control, and visual attention. However, the component loadings were unrelated to cocaine-related variables (e.g., days of use, craving), which may have been due to low variability of cocaine characteristics in the CUD group (Meade et al., 2021). DNA methylation of the NR4A1 gene has also been used as an *a priori* reference among individuals with and without temporal lobe epilepsy, and revealed components associated with differences in age of onset and poor cognitive ability (Zhi et al., 2020). These and other studies suggest multimodal fusion can elucidate the covarying structural and functional components that underlie psychiatric and neurologic conditions, and provide the framework for the current investigation.

### Paced Auditory Serial Addition Test Cognition-Associated Networks

The PASAT is a widely used measure of cognitive function, and engages a variety of processes such as working memory, sustained attention, psychomotor reaction time, and mental arithmetic. During fMRI, the PASAT activates the fronto-parietal cognitive control network (Addicott et al., 2018). In this study, PASAT cognitive performance (percent accuracy) was significantly lower among smokers compared to ex-smokers, and PASAT accuracy correlated with smoker/ex-smoker differences in fractional amplitude of low frequency fluctuation (fALFF), gray matter volume (GMV), and fractional anisotropy (FA). In particular, smokers had higher fALFF and lower GMV in the PHG, extending into the hippocampus. The group differences in cognitive accuracy reported here align with both cross-sectional and longitudinal cohort studies that have indicated slower or poorer cognitive performance among smokers compared to ex-smokers and non-smokers (Ernst et al., 2001; Starr et al., 2007; Sabia et al., 2012; North et al., 2015). Smoking-related differences in GMV have also been frequently reported. A meta-analysis of 17 voxel-based morphometry studies comparing smokers to non-smokers revealed that smokers consistently have lower GMV in the PFC and insula, and higher GMV in the lingual and occipital cortices (Yang et al., 2020). While the lower GMV may be related to smoking-accelerated atrophy, the authors speculate the regions with higher GMV may be related to attentional biases evident in tobacco use disorder (i.e., to smoking cues) (Yang et al., 2020).

However, the extent to which smoking cessation can potentially reverse or prevent cognitive-associated damage from smoking is unclear, as there are few published studies comparing smokers to ex-smokers. One such study indicated that, among older adults (aged > 67) enrolled in a smoking cessation trial, smokers who were able to maintain abstinence for 2 years had better cognitive scores (adjusted for baseline) at follow-up than smokers who did not quit (Almeida et al., 2011). The cognitive scores of smokers who did not quit worsened over the 2-year follow-up period. Compared to baseline, both smokers and ex-smokers lost GMV to a greater extent than non-smokers (Almeida et al., 2011). These results reinforce the idea that smoking cessation can prevent declines in cognitive performance, but may not prevent continued atrophy of GMV. Alternatively, the short 2-year follow-up or the advanced age of the participants may have stymied any observable recovery effects. Another small-scale study suggested that prior to making a quit attempt, smokers who were later able to maintain abstinence for 4 weeks had more GMV in the putamen and occipital lobe, and less GMV in the hippocampus and cuneus (Froeliger et al., 2010). This suggests there may be pre-existing differences among smokers that can support a quit attempt. It is possible that some of the smoker/ex-smoker differences in the present study would have been existed even if the ex-smokers had continued to smoke. However, the ex-smokers in our study quit smoking in mid-adulthood, an average of 9 years prior to the study. It is highly likely that any changes associated with smoking cessation had time to occur. To our knowledge, this is the first study linking cognitive decrements among smokers with less GMV in the PHG and hippocampus compared to long-term ex-smokers. Ultimately, a long-term longitudinal study is needed to confirm whether these results are due to the recovery from smoking-related damage rather than pre-existing group differences.

### DNA Methylation- and Abstinence Duration-Associated Networks

The duration of the most recent quit attempt was chosen as a variable because it represents the abstinence duration among the ex-smokers and provides non-zero values for the smokers, allowing for more spread in the data (all of the current smokers who reported a past quit attempt subsequently relapsed). In this study, abstinence duration was significantly lower among smokers than ex-smokers, and correlated with smoker/ex-smoker differences in fALFF, GMV, and FA. While all participants reported abstinence durations, a sub-sample also provided DNA samples for methylation analysis. Smoking reliably results in the hypomethylation of the cg05575921 loci in the *AHRR* gene in both blood and saliva (Philibert et al., 2013, 2020; Zeilinger et al., 2013; Gao et al., 2015; Ambatipudi et al., 2016), and it takes between 2 and 14 years for ex-smokers’ DNA methylation levels to return to non-smoker levels (Wilson et al., 2017; Mccartney et al., 2018). As expected, smokers had less methylation of cg05575921 than ex-smokers, and percent methylation correlated with smoker/ex-smoker differences in fALFF, GMV, and FA. Furthermore, we selected this methylation locus as a biomarker of smoking recency, as a complement to the self-reported abstinence duration. Individuals may recover from the harmful physical effects of smoking at unique rates, perhaps related their duration of smoking and smoking heaviness, in addition to other factors. Potentially, the rate of re-methylation could indicate individual differences in the rate of physiological change, which cannot be determined simply from abstinence duration. While we do not suggest that the methylation of the *AHRR* gene has any direct or causal effects specifically on brain structure or function, the associations with fALFF, GMV, and FA suggest that *AHRR* methylation may be indicative of broad, physiological changes occurring after smoking cessation. The positive correlation with abstinence duration and the overlap in methylation- and abstinence duration-associated networks supports this interpretation. Both the methylation- and abstinence duration-associated networks had overlapping patterns in fALFF LG (ex-smokers > smokers) and S/MFG (smokers > ex-smokers), as well as GMV I/MFG and insula (smokers > ex-smokers).

The pattern of FA values on different white matter tract subregions among smokers and ex-smokers differed between the methylation- and abstinence duration-associated networks. FA values are the most commonly reported diffusion measure to vary by smoking status (Gons et al., 2011; Liao et al., 2011; Hudkins et al., 2012; Lin et al., 2013; Savjani et al., 2014). The literature regarding the effects of smoking on white matter integrity is mixed, and suggests that effects vary by age. In young adulthood, smokers have reportedly higher FA values in the SLF compared to non-smokers (Liao et al., 2011). In mid-adulthood, smokers have been shown to have both lower (Lin et al., 2013; Savjani et al., 2014) and higher (Hudkins et al., 2012) FA values in the corpus callosum than non-smokers. In older adulthood, smokers, ex-smokers, and non-smokers reportedly had similar FA values in normal-appearing white matter, although smoking history was associated with higher white matter diffusivity values (Gons et al., 2011). However, the duration of smoking cessation was positively associated with higher FA values and ex-smokers with at least 20 years smoking cessation had FA values similar to non-smokers (Gons et al., 2011).

Previously, smoking epigenetic scores (representing 230 methylation loci) from 895 adults (aged 70 years) were associated with thinner cortical regions including the superior frontal and temporal cortices, as well as with poorer cognitive function and worse health (Corley et al., 2019). In Corley et al., the epigenetic scores explained a greater proportion of variance among many smoking-related morbidities than self-reported smoking histories. This suggests that individual differences in the susceptibility to, and recovery from, the detrimental effects of smoking are more strongly linked to methylation biomarkers. However, a limitation of DNA methylation as a biomarker is the cost and complexity of analysis, as well as participant concerns over privacy (thus, many participants declining to provide a sample in this study). In the future, other methylation loci that have more direct influence on brain structure and function may be identified, although it would be essential to demonstrate that the methylation of genes in blood/buccal cells is similar to those of brain cells.

### Relationship With Smoking-Related Measures

Correlations between components and other smoking-related variables indicated that the fALFF, GMV, and FA of the methylation-associated network correlated with abstinence duration and vice versa. These results suggest that the *AHRR* methylation is a complementary measure for self-reported abstinence duration. There was little overlap with the cognition-associated network, however, the fALFF component correlated with abstinence duration but no other smoking-related measures. FALFF represents regional power spectrum intensity of spontaneous oscillations in the BOLD signal while participants are at rest (Zou et al., 2008), and can suggest relative intensity of brain activity between groups. While smoker/non-smoker differences have been reported in fALFF (Wang et al., 2017; Tan et al., 2021; Weng et al., 2021), the interaction between fALFF, smoking status, and cognition is unknown. However, a growing literature has investigated the link between fALFF and cognitive impairment, and studies indicate increased fALFF values in the PHG among individuals with amnesic mild cognitive impairment (Zhang et al., 2021). This suggests a compensatory mechanism in the early stage of cognitive impairment, and may be analogous to the reduced GMV and higher fALFF values in the smokers’ PHG that correlated with cognitive performance.

## Limitations

This cross-sectional study compared current smokers to long-term ex-smokers (average 9 years abstinent). A limitation of this design is that we cannot determine whether group differences existed prior to smoking cessation. An alternative interpretation of our results is that they reflect pre-existing differences between smokers and ex-smokers; however, the overlapping methylation- and abstinence duration-associated networks strongly suggest the associated differences in WM/FA/fALFF were the result of smoking abstinence. Likewise, *AHRR* methylation positively correlated with cognitive performance, suggesting that smoking cessation can help improve cognitive performance. While a longitudinal study would provide more interpretable results, long-term (>10 years) longitudinal studies are rare and logistically complex, and shorter-term (<5 years) studies may not be long enough for measurable brain changes. For this reason, cross-sectional studies are warranted and can provide insightful, albeit indirect, information. Another limitation is that for a data-driven approach, the sample size is relatively small, although similar to Meade et al. (2021), and lacks a non-smoking control group. Lastly, while groups were matched for most demographic characteristics and smoking histories, groups differed in racial distributions.

## Conclusion

This supervised, data-driven study explored cognition/methylat ion/abstinence duration-associated fALFF + GMV + FA covarying patterns that discriminated between smokers and long-term ex-smokers. A strength of this study is the unique combination of cognitive performance, self-reported abstinence duration, as well as DNA methylation biomarkers of smoking recency. Although these cross-sectional results cannot reveal causation, our investigation suggests that quitting smoking in mid-adulthood may improve brain structure and function. Future studies should further investigate the rate and pattern of abstinence-related changes in the brains of ex-smokers compared to smokers and non-smokers, to determine the extent to which recovery from smoking effects is possible.

## Data Availability Statement

The raw data supporting the conclusions of this article will be made available by the authors upon request. The multimodal MRI and methylation data can be accessed upon request to MA, MAddicot@wakehealth.edu.

## Ethics Statement

The studies involving human participants were reviewed and approved by University of Arkansas for Medical Sciences Institutional Review Board. The patients/participants provided their written informed consent to participate in this study.

## Author Contributions

SQ performed the data analysis and wrote the manuscript. MA designed the study, collected the data, and wrote the manuscript. ZF contributed to the preprocessing of fMRI and sMRI data. LW preprocessed the dMRI data. P-CH performed the DNA methylation analysis. VC, DZ, RJ, and JS revised the manuscript. All authors contributed to the results’ interpretation and discussion and approved the final manuscript.

## Conflict of Interest

The authors declare that the research was conducted in the absence of any commercial or financial relationships that could be construed as a potential conflict of interest.

## Publisher’s Note

All claims expressed in this article are solely those of the authors and do not necessarily represent those of their affiliated organizations, or those of the publisher, the editors and the reviewers. Any product that may be evaluated in this article, or claim that may be made by its manufacturer, is not guaranteed or endorsed by the publisher.
