# Supplementary material for: Cognition, Aryl Hydrocarbon Receptor Repressor Methylation, and Abstinence Duration-Associated Multimodal Brain Networks in Smoking and Long-Term Smoking Cessation
Source: Front Neurosci. 2022 Jul 27;16:923065. doi: 10.3389/fnins.2022.923065 (PMC9363622; doi:10.3389/fnins.2022.923065)
Supplement: Supplementary file 1 [file Data_Sheet_1.docx]

Cognition, *AHRR* methylation, and abstinence duration-associated multimodal brain networks in smoking and long-term smoking cessation

**Supplementary information**

**Methods**

1. **PASAT**

A modified Paced Auditory Serial Addition Task (PASAT) [1-3] required participants to mentally sum numbers sequentially as they appeared onscreen and select the correct sum from an array of options before the next number appeared. Participants had 1.5 sec to make a response on each trial. Missed responses resulted in a “Respond Faster!” message onscreen for 1 sec. The PASAT consisted of an easy and a distress phase that were administered during functional magnetic resonance imaging. During the easy phase, two response options were shown onscreen and correct, on-time responses were followed by a neutral tone. Incorrect responses received no feedback. During the distress phase, four response options were shown onscreen and incorrect or late responses were followed by a buzzer sound. Correct, on-time responses received no feedback. In the distress phase, 55% of trials were ‘forced-fail’ trials in which the buzzer sounded regardless of the participant’s response. The easy phase was always presented before the distress phase to avoid carry-over effects of stress and negative affect. Both phases consisted of 84 trials each. The distress tolerance phase was presented immediately after the fMRI scan at a desktop computer. This task was administered as part of a larger study on the effects of smoking status on distress tolerance; the neuroimaging and distress tolerance phase data will be presented elsewhere. The primary dependent variable was the percent correct responses during the easy and distress phases.

1. **Salivary DNA methylation profiles**

Saliva samples were obtained using the Oragene-Discover kit OGR-500 (DNA Genotek, Ottawa, Ontario, Canada). Samples were prepped using the manufacturer’s protocol (PrepIT-L2P Oragene DNA Genotek Inc Kanata, Ontario, Canada). In brief, samples were centrifuged and incubated to extract DNA. DNA was quantitated using the Quant-iT PicoGreen ds DNA reagent kit (Invitrogen/Fisher Scientific, Hanover Park, IL) using a SpectraMax M5 plate reader (Molecular Devices, SunnyVale, CA). DNA concentrations were determined with the Qubit 1X dsDNA Assay Kit (catalog number Q33230, Invitrogen, Waltham, MA). gDNA was bisulfite converted with the EZ-96 DNA Methylation Kit (catalog number D5004, Zymo Research, Irvine, CA) and processed following the Infinium HD Assay Methylation Protocol (Illumina, San Diego, CA) with automated X-Stain procedures on a Tecan Freedom EVO robotic liquid handler (Zurich, CH) in the Genomics Core Facility at UAMS. Methylation EPIC beadchip arrays were scanned using an Illumina iScan. The Methylation EPIC BeadChip covers over 850,000 CpG sites, and has increased genome coverage of regulatory regions and higher reproducibility and reliability compared to previous versions [4]. Whole genome amplification, hybridization, staining and scanning steps for all samples were performed, the Illumina iScan SQ scanner was used to create images of the single arrays, and the intensities of the images were extracted using the Methylation module (v.1.9.0) of the GenomeStudio (v.2011.1) software (Illumina, San Diego, CA). Raw intensity data as IDAT files were imported into GenomeStudio for the computation of detection *p*-value of the probes, and all further steps including data import, normalization, filtering and analyses were performed using the methylation pipeline in Partek Genomics Suite^TM^ 6.6 (Partek Inc., St. Louis, MO).

1. **Methylation Data Analysis**

Percent methylation values for each CpG site (β-values) and logit-transformed ratios of methylated to unmethylated probe intensities (M-values) were extracted for further analysis [5]. For pattern identification in DNA methylation, unsupervised analysis including unsupervised hierarchical clustering [6] and Principal Component Analysis (PCA) were used. T-tests and chi-square (X2) tests were performed to evaluate differences between smokers and ex-smokers. Analysis of covariance (ANCOVA) adjusting for race, age, and gender with Fisher's Least Significant Difference contrast method were used to assess the differentially methylated CpG sites univariately. The resulting P-values were corrected for multiple comparisons using the false discovery-rate (FDR) procedure of Benjamini and Hochberg [7], with a significance threshold of P-value <0.05. Classical Receiver Operating Characteristic (ROC) curve analysis was used to evaluate the performance of a single CpG site as a biomarker of smoking status.

# **Multimodal imaging parameters**

The resting-state functional magnetic resonance images (rs-fMRI), structural MRI (sMRI) and diffusion MRI (dMRI) data were collected on a Philips 3T Achieva X-series MRI scanner (Philips Healthcare, Eindhoven, The Netherlands) with a 32-channel head coil. The rs-fMRI was acquired using single-shot echo-planar imaging (EPI) sequence. The rs-fMRI parameters were: TR/TE/FA = 2000 ms/30 ms/80°, FOV = 240 × 240 mm, matrix = 80 × 80, 37 oblique axial slices aligned with the AC-PC line, ascending sequential slice acquisition, slice thickness = 3 mm with 1 mm gap, final resolution 3.0 × 3.0 × 3.0 mm^3^. The rs-fMRI consisted of 240 volumes (8 minute duration). For the resting scan, participants were asked to remain awake with their eyes open and to focus their gaze on a fixation cross. The first four image volumes of the functional scan were discarded to allow for stabilization of the MR signal. sMRI was acquired using a high-resolution T1-weighted magnetization-prepared rapid acquisition with gradient echo (MPRAGE) sequence. The sMRI parameters were: TR/TE/FA = 8.0844/3.7010/8°, matrix = 256 × 256, 220 sagittal slices, final resolution = 0.94 × 0.94 × 1 mm^3^. dMRI was acquired using a single-shot EPI sequence. The dMRI parameters were: TR/TE/FA = 6.4/0.07/90°, matrix = 128 x 128, FOV = 224 x 224 mm, 60 axial slices, 2.5 mm thickness, no gap, final resolution = 1.75 x 1.75 x 2.5 mm^3^. Diffusion gradients were applied along 32 axes using b values of 800 s/mm^2^.

# **Preprocessing of multimodal imaging**

For fMRI, standard preprocessing based on statistical parametric mapping (SPM12, http://www.fil.ion.ucl.ac.uk/spm/) in the MATLAB 2016 environment included the following: 1) slice timing correction; 2) realignment; 3) normalization to the EPI template with 3 $\times$ 3 $\times$ 3mm^3^ resolution; 4) spatial smoothing using a 6-mm full width half-maximum Gaussian kernel; 5) nuisance covariates (6 head motions + cerebrospinal fluid [CSF] + white matter [WM]) + global signal regressed out via a general linear model from the voxel time series; and 6) calculation of fractional amplitude of low frequency fluctuations (fALFF).

For sMRI, the structural images were segmented into gray matter (GM), WM, and CSF using modulated normalization algorithms in SPM12, resulting in gray matter volume (GMV) output. Then the GMV was smoothed using a Gaussian kernel with a full width at half maximum (FWHM) = 6 mm.

For dMRI, data were preprocessed using the FMRIB Software Library v6.0.4 ([www.fmrib.ox.ac.uk/fsl](http://www.fmrib.ox.ac.uk/fsl)) and included the following: 1) simultaneous eddy current and head motion correction, along with detection of susceptibility artifacts and replacement of any outlier slices by non-parametric Gaussian Process predictions, all using the new *eddy* [1,2]; 2) diffusion tensor estimation and scalar statistics calculated by *dtifit*; 3) fractional anisotropy (FA) values were normalized to the FMRIB58 template with 1x1x1 mm^3^ resolution using Advanced Normalization Tools [3]. Finally, FA maps were resliced to 3 $\times$ 3 $\times$ 3mm^3^, and smoothed using a Gaussian kernel with FWHM = 6 mm.

After preprocessing, three representative MRI measures (fALFF from fMRI, GM volume (GMV) from sMRI, and FA from dMRI) were extracted. Next, each modality was reshaped into a feature matrix with columns representing voxels and rows representing subjects. Fusion analyses were performed with and without age, race, years of education and mean FD regressed out of the fALFF/GM/FA feature matrix. Finally, the three feature matrixes were normalized to have the same average sum of squares (computed across all subjects and all voxels for each modality) to ensure all modalities had the same range of values.

Head motion was addressed by removing outlier subjects who had micro motion (such as FD exceeding 1 mm), as well as head motion exceeding 2.0 mm of maximal translation (in any direction of x, y or z) or 1.0^o^ of maximal rotation throughout the course of scanning. Moreover, the fusion analysis was conducted on the spatial maps of fALFF, but not the original 4D fMRI data, thus micro-motion is unlikely to affect the current results.

**6. Correction for race**

The group differences in all 3 networks remained significant after including race as a covariate: 1) Cognition-associated network (for fALFF, *p* = 3.1e-04*; for GM, *p* = 0.02; for FA, *p* = 0.02), 2) Methylation-associated network (for fALFF, *p* = 4.7e-12*; for GM, *p* = 2.6e-09*; for FA, *p* = 1.2e-10*), and 3) Abstinence duration-associated network (for fALFF, *p* = 5.1e-05*; for GM, *p* = 2.1e-05*; for FA, *p* = 3.5e-05*).

**Supplementary Table 1.** Anatomical information of the identified **cognition-associated network**.

| **fALFF_IC Area** | **Right/**  **Left** | **Brodmann Area** | **volume (cc) R/L** | **random effects: Max Value (x, y, z) R/L** |
| --- | --- | --- | --- | --- |
| **Ex-smokers > smokers** |  |  |  |  |
| Posterior Cingulate Cortex | R&L | 23, 29, 30, 31 | 1.3/1.5 | 3.6 (3, -46, 22)/3.9 (0, -45, 24) |
| Middle Temporal Gyrus | R&L | 19, 21, 22, 37, 39 | 1.0/1.7 | 2.8 (56, -58, 0)/3.5 (-42, -75, 20) |
| **Smokers > ex-smokers** |  |  |  |  |
| Superior/Middle Frontal Gyrus | R&L | 6, 8, 9, 10, 45, 46, 47 | 3.8/4.0 | 3.2 (21, 34, 45)/2.8 (-6, 48, 31) |
| Parahippocampal Gyrus | R&L | 19, 30, 36, 37 | 1.3/1.0 | 3.0 (24, -46, 5)/2.8 (-27, -47, -3) |
| Lingual Gyrus | R&L | 18, 19 | 0.5/1.5 | 2.3 (9, -85, -6)/3.0 (-21, -67, -2) |
| Anterior Cingulate | R&L | 32, 33 | 1.5/0.6 | 2.7 (9, 13, 24)/2.4 (-3, 49, -2) |
| **GM_IC Area** |  | **Brodmann Area** | **volume (cc) R/L** | **random effects: Max Value (x, y, z) R/L** |
| **Ex-smokers > smokers** |  |  |  |  |
| Lingual Gyrus | R&L | 17, 18, 19 | 5.2/3.2 | 4.8 (12, -79, -4)/4.0 (-33, -70, -9) |
| Fusiform Gyrus | R&L | 18, 19, 20, 37 | 3.1/3.6 | 3.7 (36, -56, -7)/4.5 (-33, -67, -7) |
| Superior/Middle Temporal Gyrus | R&L | 21, 22, 37, 38, 39, 41, 42 | 4.6/4.7 | 4.5 (45, -49, 13)/4.2 (-45, -52, 16) |
| Parahippocampal Gyrus | R&L | 19, 28, 30, 34, 35, 36 | 3.0/3.5 | 3.1 (39, -33, -14)/4.4 (-39, -33, -11) |
| **FA_IC Area** |  | **Vol(cc)(L/R)** | **percentage** | **Zmax** |
| **Ex-smokers > smokers** |  |  |  |  |
| Forceps major | R&L | 18.2/25.1 | 32%/50% | 3.7(23,55,29)/4.2(32,10,26) |
| **Smokers > ex-smokers** |  |  |  |  |
| Superior Longitudinal Fasciculus | R&L | 35.5/31.4 | 38%/31% | 8.4(17,38,26)/4.7(32,33,37) |
| Anterior Thalamic Radiation | R&L | 22.8/25.7 | 47%/49% | 6.1(21,35,24)/6.1(32,37,16) |

**Supplementary Table 2.** Anatomical information of the identified **methylation-associated network**.

| **fALFF_IC Area** | **Right/**  **Left** | **Brodmann Area** | **volume (cc) R/L** | **random effects: Max Value (x, y, z) R/L** |
| --- | --- | --- | --- | --- |
| **Ex-smokers > smokers** |  |  |  |  |
| Lingual Gyrus | R&L | 18, 19 | 3.2/2.4 | 4.9 (24, -79, -9)/3.2 (-12, -82, -1) |
| Posterior Cingulate Cortex | R&L | 23, 30, 31 | 2.4/2.8 | 3.4 (18, -55, 6)/4.2 (0, -48, 22) |
| Fusiform Gyrus | R&L | 18, 19, 37 | 2.0/2.0 | 3.3 (21, -79, -14)/3.1 (-30, -82, -14) |
| Insula | R&L | 13 | 0.4/1.1 | 2.4 (50, -34, 18)/2.5 (-45, 3, 0) |
| **Smokers > ex-smokers** |  |  |  |  |
| Superior/Middle Temporal Gyrus | R&L | 13, 21, 22, 37, 39, 41, 42 | 1.6/4.5 | 2.8 (65, -40, 19)/3.2 (-53, -35, 7) |
| Anterior Cingulate | R&L | 24, 25, 32, 33 | 1.2/1.0 | 2.8 (9, 13, 24)/2.3 (-3, 13, 24) |
| Middle Frontal Gyrus | R&L | 6, 9, 10, 46 | 1.9/2.6 | 2.7 (36, 42, 15)/2.7 (-33, 31, 34) |
| Caudate | R&L |  | 0.9/0.6/ | 2.4 (9, 12, 8)/2.4 (-9, 1, 17) |
| Parahippocampal Gyrus | R&L | 19, 35, 36 | 0.5/0.4 | 2.0 (24, -15, -12)/2.1 (-33, -41, -3) |
| **GM_IC Area** |  | **Brodmann Area** | **volume (cc) R/L** | **random effects: Max Value (x, y, z) R/L** |
| **Ex-smokers > smokers** |  |  |  |  |
| Lingual Gyrus | R&L | 17, 18, 19 | 4.4/2.0 | 5.0 (18, -84, 4)/3.9 (-33, -70, -7) |
| Fusiform Gyrus | R&L | 18, 19, 37 | 1.0/1.2 | 2.6 (33, -38, -11)/4.5 (-45, -41, -11) |
| Superior/Middle Frontal Gyrus | R&L | 6, 8, 9, 10, 11 | 3.5/2.5 | 3.9 (27, 26, 51)/4.2 (-21, 18, 60) |
| Thalamus | R&L |  | 1.5/1.3 | 2.3 (12, -17, 9)/2.8 (-9, -14, 12) |
| Caudate | R |  | 0.1/NaN | 1.9 (15, 21, 4)/NaN |
| **Smokers > ex-smokers** |  |  |  |  |
| Middle/Inferior Frontal Gyrus | R&L | 6, 8, 9, 10, 11, 44, 45, 46, 47 | 5.0/5.3 | 4.3 (36, 25, 29)/3.8 (-33, 28, 26) |
| Anterior Cingulate | R&L | 32 | 0.3/0.7 | 2.1 (3, 47, -2)/3.0 (-3, 47, -2) |
| Insula | R&L | 13, 41 | 2.7/1.2 | 2.6 (45, -31, 21)/2.4 (-45, -25, 18) |
| **FA_IC Area** |  | **Vol(cc)(R/L)** | **percentage** | **Zmax** |
| **Ex-smokers > smokers** |  |  |  |  |
| Forceps major | R&L | 22.4/18.6 | 33%/45% | 2.7(32,56,26)/4.2(32,8,17) |
| **Smokers > ex-smokers** |  |  |  |  |
| Anterior thalamic radiation | R&L | 22/21.4 | 44%/42% | 5.6(24,34,23)/5.1(33,32,39) |
| Superior longitudinal fasciculus | R&L | 34.5/35.6 | 38%/34% | 6.9(17,40,30)/5(38,24,35) |

**Supplementary Table 3.** Anatomical information of the identified **abstinence duration-associated network**.

| **fALFF_IC Area** | **Right/**  **Left** | **Brodmann Area** | **volume (cc) R/L** | **random effects: Max Value (x, y, z) R/L** |
| --- | --- | --- | --- | --- |
| **Ex-smokers > smokers** |  |  |  |  |
| Lingual Gyrus | R&L | 18, 19 | 2.6/2.0 | 4.1 (21, -79, -9)/2.3 (-18, -47, 0) |
| Middle Frontal Gyrus | R&L | 6, 8, 9, 10, 46, 47 | 4.7/4.0 | 3.9 (39, 12, 57)/3.6 (-50, 20, 40) |
| **Smokers > ex-smokers** |  |  |  |  |
| Superior/Middle Temporal Gyrus | R&L | 13, 19, 21, 22, 38, 39, 41, 42 | 2.1/7.2 | 2.4 (45, -34, 10)/3.6 (-65, -34, 13) |
| Lingual Gyrus | R&L |  | 0.2/1.2 | 1.9 (21, -70, 1)/3.4 (-21, -78, 4) |
| Parahippocampal Gyrus | R&L | 19 | 0.2/1.3 | 1.9 (27, -49, 5)3.3 (-30, -44, -3) |
| Thalamus | R&L |  | 0.4/0.4 | 3.0 (9, -11, 17)/3.0 (-6, -14, 17) |
| **GM_IC Area** |  | **Brodmann Area** | **volume (cc) R/L** | **random effects: Max Value (x, y, z) R/L** |
| **Smokers > ex-smokers** |  |  |  |  |
| Inferior/Middle Frontal Gyrus | R&L | 6, 8, 9, 10, 44, 46, 47 | 5.4/4.5 | 3.8 (45, 4, 27)/3.2 (-39, 29, -4) |
| Insula | R&L | 13, 41, 47 | 2.2/1.3 | 2.1 (42, 17, -1)/1.9 (-56, -34, 18)/ |
| **FA_IC Area** |  | **Vol(cc)(R/L)** | **percentage** | **Zmax** |
| **Ex-smokers > smokers** |  |  |  |  |
| Anterior thalamic radiation | R&L | 20.1/17.1 | 35%/38% | 7.1(19,39,27)/6.2(31,40,18) |
| Superior longitudinal fasciculus | R&L | 34.3/36.1 | 38%/33% | 7.4(18,39,22)/4.3(36,18,35) |
| **Smokers > ex-smokers** |  |  |  |  |
| Forceps major | R&L | 22.7/17.8 | 31%/45% | 3.2(29,45,24)/3(20,13,26) |

References

1 Lejuez CW, Kahler CW, Brown RA. A modified computer version of the Paced Auditory Serial Addition Task (PASAT) as a laboratory-based stressor. Behav Ther. 2003;26:290-93.

2 Daughters SB, Ross TJ, Bell RP, Yi JY, Ryan J, Stein EA. Distress tolerance among substance users is associated with functional connectivity between prefrontal regions during a distress tolerance task. Addict Biol. 2016;22:1378-90.

3 Addicott MA, Daughters SB, Strauman TJ, Appelbaum LG. Distress tolerance to auditory feedback and functional connectivity with the auditory cortex. Psychiatry Res Neuroimaging. 2018;282:1-10.

4 Pidsley R, Zotenko E, Peters TJ, Lawrence MG, Risbridger GP, Molloy P, et al. Critical evaluation of the Illumina MethylationEPIC BeadChip microarray for whole-genome DNA methylation profiling. Genome biology. 2016;17(1):208.

5 Du P, Zhang X, Huang CC, Jafari N, Kibbe WA, Hou L, et al. Comparison of Beta-value and M-value methods for quantifying methylation levels by microarray analysis. BMC Bioinformatics. 2010;11:587.

6 Quackenbush J. Computational analysis of microarray data. Nat Rev Genet. 2001;2(6):418-27.

7 Benjamini Y, Hochberg Y. Controlling the False Discovery Rate - a Practical and Powerful Approach to Multiple Testing. J R Stat Soc B. 1995;57(1):289-300.

8 Addicott MA, Luber B, Nguyen D, Palmer H, Lisanby SH, Appelbaum LG. Low- and High-Frequency Repetitive Transcranial Magnetic Stimulation Effects on Resting-State Functional Connectivity Between the Postcentral Gyrus and the Insula. Brain Connect. 2019;9(4):322-28.
